# Supplementary material for: Modelling vegetation understory cover using LiDAR metrics
Source: PLoS One. 2019 Nov 27;14(11):e0220096. doi: 10.1371/journal.pone.0220096 (PMC6881062; doi:10.1371/journal.pone.0220096)
Supplement: S1 Table — (DOCX) [file pone.0220096.s001.docx]

| Code | Name | Definition |
| --- | --- | --- |
| GAP | Gap Fraction | No. of Returns in a vertical bin divided by all returns in and below the vertical bin for the plot (12 m radius circle) |
| LAD | Leaf Area Density | -log(Gap Fraction)/(k*dz), where k =0.5 (extinction coefficient), and dz=0.5 m (the bottom limit of the profile which we set to 0.5 m) for the plot |
| STRATUM | Vertical Stratum | Three vertical bins (0.5 m-1.5 m, 1.5 m-2.5 m, 2.5 m-3.5 m) |
| OVERSTORY | Overstory | Crown closure (CC) above the stratum of interest divided into three classes (low, medium, high) |
| VOX1m | Voxel 1 m^3^ cover | Number of occupied 1 m^3^ voxels in the Stratum |
| VOX50cm | Voxel 50 cm^3^ cover | Number of occupied 50 cm^3^ voxels in the Stratum |
| NORM | Normalized Cover | No. of Returns in a Stratum divided by first returns in the column |
| FRAC | Fractional Cover | No. of Returns in a Stratum divided by ground returns and vertical bin returns |
| CC | Crown Closure | Proportion of the plot area (12 m radius circle) that is occupied in a stratum based on data filtered using a 0.25 x 0.25 pixel. |
| TYPE | Forest Type | One of 4 forest types identified in the Petawawa Research Forest; Pine (P), Pine Mixedwood (X), Red Oak(R), Mixedwood (M) |
| SLICE | Vertical slice returns | No. of Returns in a Stratum divided by all returns in the column |
| AVG_CANOPY_HT | Average Canopy Height |  |
| VAR_CANOPY_HT | Variance of Canopy Height | Variance in canopy height within plot. |
| LAD.OS | Overstory estimated using LAD metric | The LAD value in the column above the stratum of interest |
| GAP.OS | Overstory estimated using GAP metric | The GAP value in the column above the stratum of interest |
| CC.OS | Crown closure Overstory | The CC value in the column above the stratum of interest |
| VOX1m.OS | Overstory based on VOX1m data | Number of occupied 1 m^3^ voxels above the stratum |
| VOX50cm.OS | Overstory based on NORM calculation | Number of occupied 50 cm^3^ voxels above the stratum |
| NORM.OS | Overstory based on NORM calculation | The NORM value in the column above the stratum of interest |
| FRAC.OS | Overstory based on FRAC calculation | The FRAC value in the column above the stratum of interest |
| SLICE.OS | Overstory based on SLICE calculation | The SLICE value in the column above the stratum of interest |
| VCI | Vertical Complexity Index | Index of vertical vegetation complexity |
| P90 | Canopy Height | 90^th^ percentile of canopy height |
| GAP_01 (40) | GAP value for 0.5 m to 1.5 m bin | GAP value for each of 40-1 m vertical bins starting at 0.5 m |
| LAD_01 (40) | GAP value for 0.5 m to 1.5 m bin | GAP value for each of 40-1 m vertical bins starting at 0.5 m |
| FRAC_01 (40) | FRAC value for 0.5 m to 1.5 m bin | FRAC value for each of 40-1 m vertical bins starting at 0.5 m |
| NORM_01 (40) | NORM value for 0.5 m to 1.5 m bin | NORM value for each of 40-1 m vertical bins starting at 0.5 m |
| VOX1m_01 (40) | VOX1m value for 0.5 m to 1.5 m bin | VOX1m value for each of 40-1 m vertical bins starting at 0.5 m |
| VOX50cm_01 (40) | VOX 50 cm value for 0.5 m to 1.5 m bin | VOX50cm value for each of 40-1 m vertical bins starting at 0.5 m |
| CC_01 (40) | CC value for 0.5 m to 1.5 m bin | CC value for each of 40-1 m vertical bins starting at 0.5 m |
| SLICE_01 (40) | SLICE value for 0.5 m to 1.5 m bin | SLICE value for each of 40-1 m vertical bins starting at 0.5 m |
