# Supplementary material for: Modelling vegetation understory cover using LiDAR metrics
Source: PLoS One. 2019 Nov 27;14(11):e0220096. doi: 10.1371/journal.pone.0220096 (PMC6881062; doi:10.1371/journal.pone.0220096)
Supplement: S2 Table — (DOCX) [file pone.0220096.s002.docx]

| Rank | Variable |  |
| --- | --- | --- |
| 1 | VOX1m | |
| 2 | LAD | |
| 3 | GAP | |
| 4 | NORM | |
| 5 | STRATUM | |
| 6 | CC | |
| 7 | VOX50cm | |
| 8 | SLICE | |
| 9 | FRAC | |
| 10 | VOX1m_01 | |
| 11 | GAP_02 | |
| 12 | LAD_02 | |
| 13 | FRAC_03 | |
| 14 | VOX1m_02 | |
| 15 | CC_02 | |
| 16 | VOX50cm_02 | |
| 17 | VOX50cm_01 | |
| 18 | LAD_01 | |
| 19 | SLICE_02 | |
| 20 | FRAC_02 | |
| 21 | FRAC_01 | |
| 22 | NORM_01 | |
| 23 | VOX50cm_03 | |
| 24 | CC_01 | |
| 25 | LAD.OS | |
| 26 | NORM_03 | |
| 27 | VOX1m_03 | |
| 28 | CC_03 | |
| 29 | LAD_03 | |
| 30 | SLICE.OS | |
| 31 | GAP_03 | |
| 32 | SLICE_03 | |
| 33 | FRAC.OS | |
| 34 | CC.OS | |
| 35 | NORM.OS | |
| 36 | VOX50cm_05 | |
| 37 | VOX1m.OS | |
| 38 | AVG_CANOPY_HT | |
| 39 | LAD_05 | |
| 40 | VOX50cm.OS | |
| 41 | GAP_05 | |
| 42 | GAP_06 | |
| 43 | GAP_09 | |
| 44 | GAP_10 | |
| 45 | FRAC_06 | |
| 46 | GAP_12 | |
| 47 | NORM_06 | |
| 48 | VOX50cm_08 | |
| 49 | FRAC_08 | |
| 50 | VOX1m_09 | |
| 51 | SLICE_08 | |
| 52 | FRAC_12 | |
| 53 | FRAC_18 | |
| 54 | FRAC_15 | |
| 55 | FRAC_16 | |
| 56 | NORM_16 | |
| 57 | VOX50cm_10 | |
| 58 | GAP.OS | |
| 59 | CC_12 | |
