# Supplementary material for: Modelling vegetation understory cover using LiDAR metrics
Source: PLoS One. 2019 Nov 27;14(11):e0220096. doi: 10.1371/journal.pone.0220096 (PMC6881062; doi:10.1371/journal.pone.0220096)
Supplement: S3 Table — (DOCX) [file pone.0220096.s003.docx]

| Predictor | # of times in top 5 of model | # of times in top 10 of model |
| --- | --- | --- |
| GAP | 18 | 18 |
| STRATUM | 18 | 18 |
| VOX1 | 16 | 18 |
| LAD | 15 | 16 |
| FRAC | 2 | 13 |
| NORM | 6 | 11 |
| CC | 3 | 11 |
| GAP_02 | 4 | 9 |
| CC_01 | 4 | 7 |
| VOX50 | 0 | 9 |
| SLICE | 0 | 7 |
| LAD_03 | 0 | 7 |
| LAD_02 | 0 | 5 |
| AVG_CANOPY_HT | 0 | 4 |
