# Supplementary material for: Modelling vegetation understory cover using LiDAR metrics
Source: PLoS One. 2019 Nov 27;14(11):e0220096. doi: 10.1371/journal.pone.0220096 (PMC6881062; doi:10.1371/journal.pone.0220096)
Supplement: Attachment — Submitted filename: PONE-D-19-19180_reviewer_comments.docx [file pone.0220096.s005.docx]

**PLOS ONE – Manuscript ID: PONE-D-19-19180**

**Title: Modelling vegetation understory cover using LiDAR metrics**

**Reviewer comments:**

**Overall comments:**

This is a well-written paper with logical flow that is easy to follow, has sufficient detail for replication by others, and use of terminology and acronyms is appropriate for audience. It is obvious a lot of hard work went into this project, and this paper does a great job of explaining it. I am especially impressed by the thorough lit review and excellent figures and tables, which some authors don’t put a lot of effort into. I have a few very minor comments, below.

**Abstract**

Line 27: has yet to be fully validated.

**Introduction**

Line 86 (and as it occurs thereafter): some papers cite Random Forests with capital letters. I think it is fine either way.

Excellent lit review. The authors did an especially good job exploring various options for analysis.

**Methods**

Line 126: extra space between composition features

Thorough Methods section. I like Figure 1.

**Analysis**

Line 214: insert space between variables (26)

Line 229: insert space between package (18…

Thorough presentation of analysis methods.

**Results**

Solid statistical analysis methods and presentation of results.

There are a lot of table but I’m not sure how to suggest minimizing them as they all contain pertinent info to the study.

_________________________________

Overall references look good, but I did not take a close look at each of them.

Figures and Tables look great!
